# The footprint of the ageing stroma in older patients with breast cancer

**DOI:** 10.1186/s13058-017-0871-0

**Published:** 2017-07-03

**Authors:** Barbara Brouwers, Debora Fumagalli, Sylvain Brohee, Sigrid Hatse, Olivier Govaere, Giuseppe Floris, Kathleen Van den Eynde, Yacine Bareche, Patrick Schöffski, Ann Smeets, Patrick Neven, Diether Lambrechts, Christos Sotiriou, Hans Wildiers

**Affiliations:** 1 Laboratory of Experimental Oncology (LEO), Department of Oncology, KU Leuven, Leuven, Belgium; 2 Department of General Medical Oncology, Leuven Cancer Institute, University Hospitals Leuven, Leuven, Belgium; 3 Breast Cancer Translational Research Laboratory, Institut Jules Bordet, Universite Libre de Bruxelles, Brussels, Belgium; 4 Department of Imaging and Pathology, Laboratory of Translational Cell & Tissue Research, KU Leuven, Herestraat 49, B-3000 Leuven, Belgium; 5 Department of Pathology, University Hospitals Leuven, Herestraat 49, B-3000 Leuven, Belgium; 6 Multidisciplinary Breast Center, University Hospitals Leuven, Leuven, Belgium; 7 Department of Oncology, Laboratory for Translational Genetics, Vesalius Research Center (VRC), Vlaams Instituut voor Biotechnologie (VIB) and KU Leuven, Leuven, Belgium

**Keywords:** Breast cancer, Stroma, Senescence, Senescence-associated secretory profile, Autophagy, Gene expression, Old patients, Ageing

## Abstract

**Background:**

Tumours are not only composed of malignant cells but also consist of a stromal micro-environment, which has been shown to influence cancer cell behaviour. Because the ageing process induces accumulation of senescent cells in the body, this micro-environment is thought to be different in cancers occurring in old patients compared with younger patients. More specifically, senescence-related fibroblastic features, such as the senescence-associated secretory profile (SASP) and the induction of autophagy, are suspected to stimulate tumour growth and progression.

**Methods:**

We compared gene expression profiles in stromal fields of breast carcinomas by performing laser capture microdissection of the cancer-associated stroma from eight old (aged ≥80 years at diagnosis) and nine young (aged <45 years at diagnosis) patients with triple-negative breast cancer. Gene expression data were obtained by microarray analysis (Affymetrix). Differential gene expression and gene set enrichment analysis (GSEA) were performed.

**Results:**

Differential gene expression analysis showed changes reminiscent of increased growth, de-differentiation and migration in stromal samples of older versus younger patients. GSEA confirmed the presence of a SASP, as well as the presence of autophagy in the stroma of older patients.

**Conclusions:**

We provide the first evidence in humans that older age at diagnosis is associated with a different stromal micro-environment in breast cancers. The SASP and the presence of autophagy appear to be important age-induced stromal features.

**Electronic supplementary material:**

The online version of this article (doi:10.1186/s13058-017-0871-0) contains supplementary material, which is available to authorized users.

## Background

Oncological research over the past decades has been focussed primarily tumour cell characteristics. However, tumoural masses are not exclusively composed of malignant cells; they also comprise a stromal component containing endothelial cells, (myo)fibroblasts, smooth muscle cells, adipocytes and inflammatory cells. Research on the stromal component of tumour masses has shown that stromal characteristics are correlated with disease outcome and behaviour [1–10] in several malignancies. The stroma seems to play a very important role in tumour initiation, progression and metastatic spread [11, 12]. The fibroblasts contained in this stromal compartment show a specific phenotype and are called *carcinoma-associated fibroblasts* [13]. Because cellular senescence progressively occurs throughout a person’s lifetime in fibroblasts of various origins [14], it seems plausible that the characteristics of the stromal compartment of breast cancers would differ between young and older patients and that this could result in a pro-tumourigenic micro-environment with stimulation of proliferation, migration/invasion and de-differentiation.

The incidence of breast cancer, the most frequent tumour occurring in women, increases with age [15, 16]. Cancer in older patients is thought to arise from lifelong exposure to harmful stimuli, such as DNA-damaging agents, oxidative stress factors and telomeric loss. In addition, the micro-environmental changes caused by senescent cells might also be an important harmful trigger. Breast cancer in young patients usually reflects either a genetic defect or the impact of early life-transforming effects on an immature breast epithelium.

Senescence in general is a protective mechanism that shuts down damaged cells [17]. Nature has selected for this mechanism to protect young organisms from developing cancer. Senescent cells are forced into a state of irreversible growth arrest [18, 19] and exhibit a specific phenotype characterised by enlarged size, flattened morphology, senescence-associated β-galactosidase activity, reorganisation of chromatin into foci of heterochromatin and resistance to apoptosis [20]. They also acquire the so-called senescence-associated secretory profile (SASP) [21, 22], maintaining the growth arrest and recruiting immune cells towards the damaged cells in order to eradicate them. However, the SASP also seems to have a detrimental influence on nearby cells. Epithelial cells neighboured by senescent fibroblasts lose differentiated properties, become invasive and undergo full malignant transformation [20, 23–25]. In this process, a major role has been attributed to matrix metalloproteinase 3 [23] together with other components of the SASP [25, 26], such as inflammatory cytokines and chemokines. This concept of senescence as a useful cancer-protective mechanism in younger life but a detrimental cancer-promoting mechanism in later life has repeatedly been described as an example of ‘antagonistic pleiotropy’ [27, 28] in cellular or animal models [23, 24, 29–32]. Senescent cells have been reported in vivo in a variety of tissues of different organisms, including mice, primates and humans [14, 33–36]. Also, studies have provided evidence that increasing age does result in a higher frequency of senescent cells [14, 33, 34, 37], albeit mostly in the skin.

An additional mechanism that has been proposed to explain the tumour-promoting effects of a senescent micro-environment is the ‘the autophagic tumor stroma model of cancer’ [38–42]. This model states that fibroblasts, in transition to a senescent state, activate the autophagic process. During this so-called autophagy-to-senescence transition (AST), the cells shift towards an aerobic glycolysis metabolism, creating high-energy mitochondrial fuels that feed the nearby epithelial cancer cells. Autophagic fibroblasts were shown to have tumour- and metastasis-promoting activity [39]. The discovery of this concept was preceded by the finding that tumoural cells can induce AST in surrounding fibroblasts by secreting hydrogen peroxide that causes oxidative stress and activation of autophagy in the fibroblasts. This process was named the *reverse Warburg effect* (as opposed to the original idea, called the *Warburg effect*, by which aerobic glycolysis takes place in epithelial cancer cells). Fibroblasts displaying a constitutively activated autophagy programme turned out to show many morphological characteristics of senescence, including induction of *P21* ^*WAF1/CIP1*^, which led to the hypothesis that AST is one of the mechanisms by which senescent stromal cells create a ‘fertile soil’ for the initiation and progression of cancer.

Despite this knowledge, stromal differences with increasing patient age have so far never been investigated in vivo, and little clinical evidence can be found for a more aggressive behaviour of tumour cells growing in a context of ‘older’ stroma. On the contrary, breast cancers in older patients have in general been shown to grow more slowly and to behave less aggressively, even when adjusting for different histological tumour characteristics [43, 44]. On these premises, we sought to compare gene expression profiles of tumour-adjacent stroma in older versus younger patients with breast cancer matched for other clinico-pathologic parameters.

## Methods

### Patient selection and clinical specimens

This study was approved by the ethics committee of the University Hospitals Leuven (Leuven, Belgium) in accordance with the International Conference on Harmonisation Harmonised Tripartite Guideline for Good Clinical Practice. Candidate patients were selected using the following criteria: (1) aged <45 years or ≥80 years, (2) no neo-adjuvant chemotherapy treatment or hormone treatment before surgery, (3) surgery for early triple-negative breast cancer (defined as oestrogen receptor [ER] and progesterone receptor [PR] <1% and human epidermal growth factor receptor 2 [HER2] <2+ by immunohistochemistry or fluorescent in situ hybridisation-negative) with fresh frozen resection specimens available (stored at −80 °C at the pathology department of the University Hospitals Leuven) and (4) no chronic inflammatory diseases to exclude confounding variables.

**Fig. 1:**
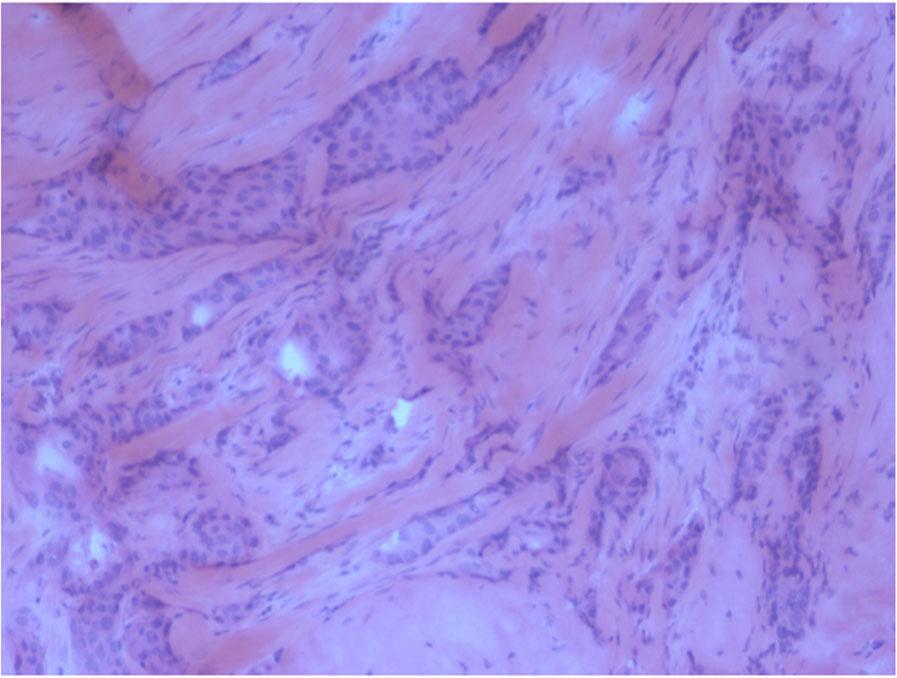
Haematoxylin and eosin-stain of selected tumour block for laser capture microdissection

For each candidate patient, one section of the frozen tumour material was obtained for hematoxylin and eosin (H&E) staining. H&E-stained sections were evaluated for tumoural and stromal content and used to localise the best areas for stromal microdissection (*see* Fig. 1 as an example). Only tumour tissue blocks consisting of invasive tumour with representative carcinoma-associated stromal fields to allow laser capture microdissection (LSM) were selected. We selected tumours with a very low amount of or absent tumour-infiltrating lymphocytes to prevent bias in the gene expression analyses. On the basis of the above criteria, 17 female patients with breast cancer (9 young patients <45 years old at diagnosis and 8 old patients ≥80 years old at diagnosis) were included in the study.

### Staining procedures and laser capture microdissection

#### Preparation of the tissue slides

For the selected patients, ten frozen sections of 10-μm thickness were mounted onto specific membrane slides (steel frames with polyethylene terephthalate membrane, catalogue number 11505151; Leica Microsystems, Wetzlar, Germany) and were kept at −80 °C until the staining and dissection procedure was started. All tissue slides underwent LCM within 7 days after preparation.

### Staining

Prior to LCM, tumour slides were stained with cresyl violet following a procedure optimised for maximising RNA yield. Briefly, tumour slides were taken from −80 °C and were fixed into a 95% ethanol solution for 30 seconds. Next, they were transferred to ethanol solutions with progressively decreasing concentrations (75%, 50%) for 30 seconds each. Then, cresyl violet dye (cresyl violet acetate pure high-purity biological stain, catalogue number AC229630050; Acros Organics, Geel, Belgium) at a concentration of 0.2% was applied for 30–60 seconds, after which dehydration of the tissue was achieved by rinsing the slides with increasing concentrations of ethanol (50%, 75%, 95%, 100%, 100%) for 15 seconds each.

### Laser capture microdissection

**Fig. 2:**
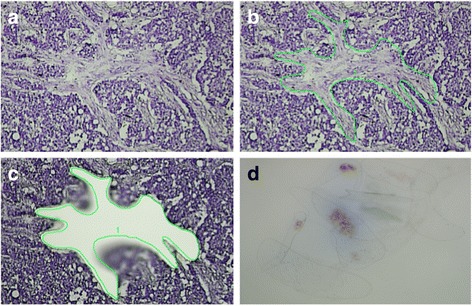
Laser capture microdissection of cancer associated stroma. **a**–**c** Microdissection procedure. **d** Yield of stromal pieces after repeated microdissection within the same tumour slide

After the staining procedure, LCM was accomplished within 30 minutes by using a laser microscope (LMD6500; Leica Microsystems). Dissected stromal pieces were immediately collected in an RNase/DNase-free capture vial containing 25 μl of stabilising RNA extraction buffer. During dissection, care was taken to avoid blood vessels, zones containing infiltrating immune cells, or fatty tissue. Dissection was restricted to fields contained within the perimeter of the invasive tumour or at the invasive front of the tumour, but in direct relationship with invasive epithelial nests. Pictures were taken before and after the dissection procedure (*see* Fig. 2 as an example). After finishing dissection for one tumour slide, 25 μl of RNA extraction buffer was added to the capture vial, and lysis was performed for 30 minutes at 42 °C. The obtained lysate was stored at −80 °C until further RNA extraction. For each patient, several tumour slides were laser-dissected using this procedure (seven to ten slides per patient according to size and amount of stromal fields within the tumour tissue).

### RNA extraction and amplification

RNA isolation was performed using the Arcturus PicoPure RNA extraction kit (PicoPure^TM^ Frozen RNA Isolation Kit, catalogue number KIT0202/KIT0204; Arcturus, Mountain View, CA, USA) according to the manufacturer’s protocol. Briefly, lysates from the same tumour were combined, and after addition of 50 μl of ethanol 70%, the pooled samples were passed onto pre-conditioned RNA extraction columns. After centrifugation and washing, DNase was applied onto the column to eliminate residual DNA (RNase-Free DNase Set, catalogue number 79254; QIAGEN, Hilden, Germany). After a washing step, the purified RNA was eluted from the column using 11 μl of elution buffer. Samples were subsequently tested for RNA quality (RNA Quality Indicator) on the Experion™ system (Bio-Rad Laboratories, Hercules, CA, USA) using high-sensitivity RNA chips, and concentrations were measured using the NanoDrop 2000 spectrophotometer (Thermo Scientific, Wilmington, DE, USA). The quality of the RNA varied between samples, which is a known limitation of the LSM procedure [45] (see Additional file 1). Prior to microarray analysis, RNA was pre-amplified using the Ovation PicoSL WTA System V2 (catalogue number 3312-24; NuGEN, Leek, The Netherlands). The Ribo-SPIA (single-primer isothermal amplification) technology implemented in this procedure is ideal for amplification of partially degraded and compromised RNA samples, contributes minimal coverage bias, and is highly reproducible [46]. The procedure is widely used in LCM projects and does not introduce significant bias into relative gene expression values [47, 48]. A clean-up step using the MinElute Reaction Cleanup Kit (catalogue number 28204; QIAGEN) was also incorporated into the amplification procedure. After NuGEN pre-amplification of the RNA samples, quantitative reverse transcription-polymerase chain reaction assessment of common housekeeping genes showed that the amplification procedure had resulted in highly concentrated complementary DNA fragments with sufficient size to be recognised by the primers (data not shown).

### Gene expression analysis

Gene expression was analysed using Human Genome U133Plus2 microarray chips (Affymetrix, Santa Clara, CA, USA) at the J.C. Heuson Breast Cancer Translational Research Laboratory (Jules Bordet Institute, Brussels, Belgium) according to the manufacturer’s instructions. Standard quality assessments were conducted on the resulting files, and all samples passed quality assurance for further analysis. Expression values were computed using the frozen robust multi-array analysis (fRMA) normalisation method (‘frma’ package in Bioconductor) [49]. When multiple probe sets mapped to the same official gene symbol, we computed their average value. The expression data are available from the Gene Expression Omnibus (GEO) repository under accession number [GEO:GSE90521].

### Statistical analysis

#### Differential expression analysis

To identify the genes that were differentially expressed in the two age categories (<45 years versus ≥80 years), we computed for each probe set the mean expression value in both age groups and calculated the fold change of these means (i.e., the ratio of the average expression of this particular gene in young and old patients). We used a Wilcoxon test. Genes with fold change greater than 1.5 or less than −1.5 were considered differentially expressed.

#### In silico validation

To validate the obtained differential gene expression, gene expression data sets from other projects in which researchers investigated laser-dissected stromal samples obtained from patients with breast cancer were retrieved. The data sets were available in the National Center for Biotechnology Information (NCBI) GEO database under accession numbers [GEO:GSE5847] [9], [GEO:GSE4823] [7] and [GEO:GSE14548] [8].

#### Gene set enrichment analysis

Gene set enrichment analysis (GSEA) was conducted using our local reimplementation of the GSEA algorithm developed at the Broad Institute [50]. Briefly, genes were ranked according to their fold change in young versus old patients, and an enrichment score (ES) ranging from −1 to 1 was computed. This score reflects to what extent the genes constituting a given reference class are enriched among the top up- or down-regulated genes of the differential expression analysis. Low (negative) ES values correspond to an enrichment of the reference class among genes that are up-regulated in old patients, whereas high (positive) ES values correspond to an enrichment of the reference class among genes that are up-regulated in young patients. The false discovery rate-adjusted *p* values associated with each ES value reflect the probability that an ES at least as high or as low could be obtained merely by chance. Adjusted *p* values <0.05 were considered significant.

## Results

### Patient demographics

For the purpose of the present study, 17 female patients (9 young patients <45 years old at diagnosis and 8 old patients aged ≥80 years at diagnosis) with available fresh frozen breast cancer resection specimens and with sufficient stroma to allow laser microdissection were selected. Extreme age categories were chosen to maximise the probability of detecting significant age-related differences. All patients underwent surgery for early breast cancer at the Multidisciplinary Breast Center (University Hospitals Leuven, Belgium) between 2000 and 2011. All patients had invasive ductal carcinomas >1.5 cm and were negative for ER, PR and HER2. Additional patient and tumour characteristics are summarised in Table 1. The choice of triple-negative breast cancers was made to exclude cancer-related confounding factors as much as possible.

**Table 1:** Patient and tumour characteristics

Patient	Age at diagnosis (years)	ER	PR	HER2	Tumour type	Tumour grade	Maximum tumour size (cm)	pT stage	pN stage
6	27	Neg	Neg	Neg	Ductal	3	2.3	2	0
5	30	Neg	Neg	Neg	Ductal	3	2.5	2	0
7	32	Neg	Neg	Neg	Ductal	3	2.2	2	0
1	33	Neg	Neg	Neg	Ductal	3	2.8	2	0
3	39	Neg	Neg	Neg	Ductal	3	3.0	2	0
2	43	Neg	Neg	Neg	Ductal	3	3.0	2	2a
4	44	Neg	Neg	Neg	Ductal	3	2.8	2	0
8	44	Neg	Neg	Neg	Ductal	3	3.5	2	0
9	44	Neg	Neg	Neg	Ductal	3	3.0	2	0
12	80	Neg	Neg	Neg	Ductal	3	4.0	2	0
16	82	Neg	Neg	Neg	Ductal	3	3.5	2	0
17	82	Neg	Neg	Neg	Ductal	3	1.5	1c	0
13	82	Neg	Neg	Neg	Ductal	2	3.0	2	3a
15	83	Neg	Neg	Neg	Ductal	3	3.8	2	1a
10	83	Neg	Neg	Neg	Ductal	3	3.2	2	0
11	86	Neg	Neg	Neg	Ductal	3	3.0	2	0
14	87	Neg	Neg	Neg	Ductal	3	2.0	1c	0

*Abbreviations: pT* pathological T stage, *pN* pathological N stage, *ER* Oestrogen receptor, *PR* Progesterone receptor, *HER2* Human epidermal growth factor receptor 2

### Differential gene expression analysis

**Fig. 3:**
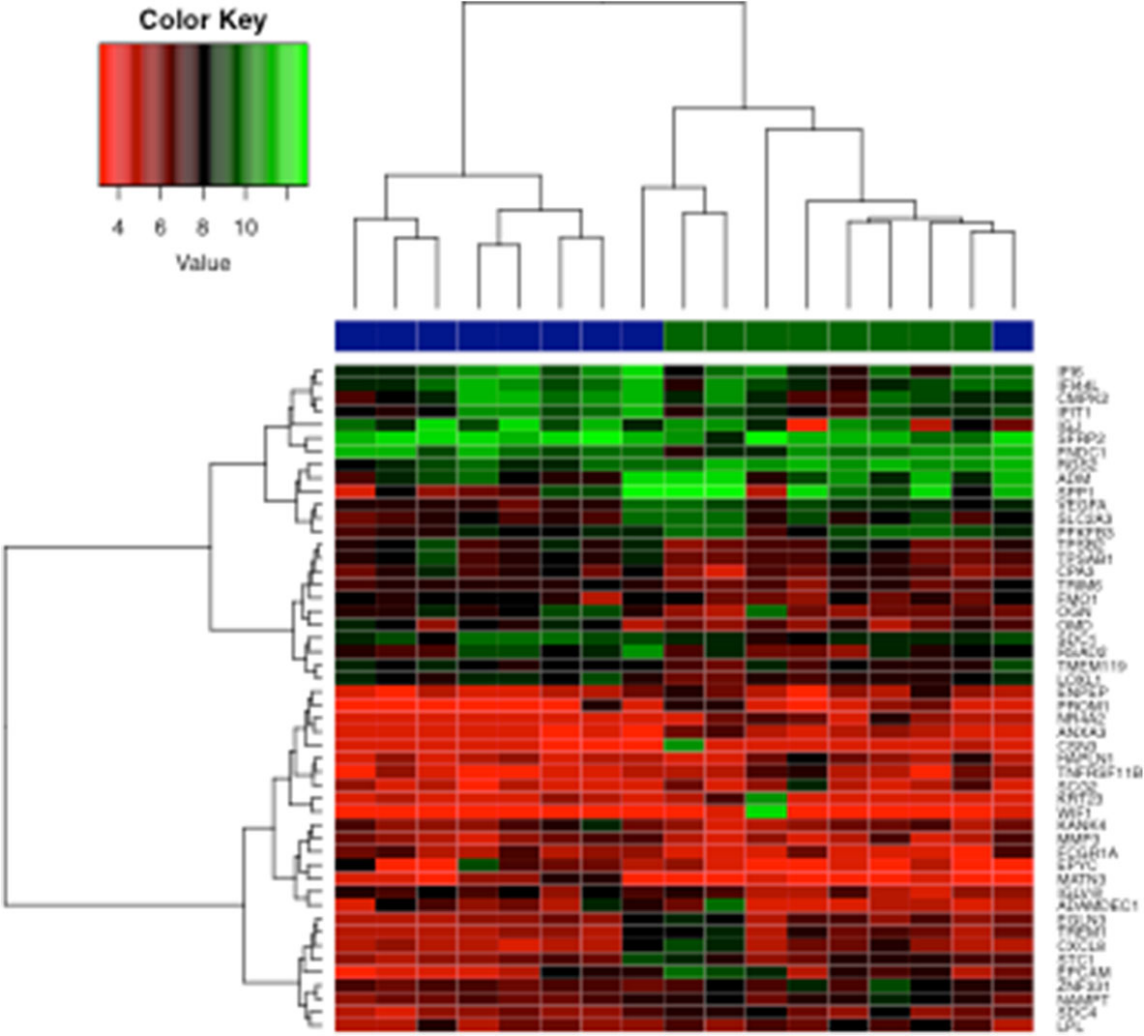
Heat maps constructed using the 25 top up-regulated and the 25 top down-regulated genes. *Blue* represents young patients, and *green* represents old patients

A differential gene expression analysis using a 1.5-fold up- or down-regulation as the cut-off revealed 120 genes that were up-regulated in older subjects’ stromal samples and 107 genes that were down-regulated in older subjects’ stromal samples compared with younger subjects (Table 2). Heat maps constructed using the 25 top up- and down-regulated genes are shown in Fig. 3.

**Table 2:** Genes with greater than 1.5-fold or less than −1.5-fold expression and respective fold changes

Gene	Full name	Fold change
*SPP1*	Secreted phosphoprotein 1	−4.79
*EPCAM*	Epithelial cell adhesion molecule	−4.02
*IL8*	Interleukin 8	−2.74
*NR4A2*	Nuclear receptor subfamily 4, group A, member 2	−2.45
*RGS2*	Regulator of G-protein signaling 2, 24 kDa	−2.41
*TREM1*	Triggering receptor expressed on myeloid cells 1	−2.36
*PROM1*	Prominin 1	−2.27
*SCG2*	Secretogranin II	−2.22
*LPL*	Lipoprotein lipase	−2.20
*SDC4*	Syndecan 4	−2.19
*SLC2A3*	Solute carrier family 2 (facilitated glucose transporter), member 3	−2.13
*PFKFB3*	6-Phosphofructo-2-kinase/fructose-2,6-biphosphatase 3	−2.11
*TNFRSF11B*	Tumour necrosis factor receptor superfamily, member 11b	−2.11
*WIF1*	WNT inhibitory factor 1	−2.10
*NAMPT*	Nicotinamide phosphoribosyltransferase	−2.08
*ENPEP*	Glutamyl aminopeptidase (aminopeptidase A)	−2.07
*ZNF331*	Zinc finger protein 331	−2.07
*ANXA3*	Annexin A3	−2.06
*HAPLN1*	Hyaluronan and proteoglycan link protein 1	−2.05
*CSN3*	Casein kappa	−2.05
*KRT23*	Keratin 23 (histone deacetylase inducible)	−2.05
*VEGFA*	Vascular endothelial growth factor A	−2.03
*STC1*	Stanniocalcin 1	−2.01
*EGLN3*	Egl nine homolog 3 (*C. elegans*)	−1.97
*ADM*	Adrenomedullin	−1.96
*G0S2*	G0/G1 switch 2	−1.95
*BAMBI*	BMP and activin membrane-bound inhibitor homolog (*Xenopus laevis*)	−1.93
*TDO2*	Tryptophan 2,3-dioxygenase	−1.93
*CD24*	CD24 molecule	−1.92
*DNER*	Delta/Notch-like epidermal growth factor-related receptor	−1.92
*IBSP*	Integrin-binding sialoprotein	−1.91
*HSPA2*	Heat shock 70 kDa protein 2	−1.90
*ERRFI1*	ERBB receptor feedback inhibitor 1	−1.89
*MUCL1*	Mucin-like 1	−1.89
*APOLD1*	Apolipoprotein L domain containing 1	−1.89
*SHISA2*	Shisa homolog 2 (*Xenopus laevis*)	−1.88
*GPX3*	Glutathione peroxidase 3 (plasma)	−1.87
*SERPINE1*	Serpin peptidase inhibitor, clade E (nexin, plasminogen activator inhibitor type 1), member 1	−1.87
*COL2A1*	Collagen, type II, α 1	−1.86
*CP*	Ceruloplasmin (ferroxidase)	−1.85
*COL9A3*	Collagen, type IX, α 3	−1.85
*ENO2*	Enolase 2 (gamma, neuronal)	−1.84
*FOSB*	FBJ murine osteosarcoma viral oncogene homolog B	−1.84
*TSPAN13*	Tetraspanin 13	−1.82
*CYP4X1*	Cytochrome P450, family 4, subfamily X, polypeptide 1	−1.82
*TFAP2C*	Transcription factor AP-2γ (activating enhancer binding protein 2γ)	−1.81
*EGR3*	Early growth response 3	−1.81
*SOX11*	SRY (sex-determining region Y), box 11	−1.79
*CLEC5A*	C-type lectin domain family 5, member A	−1.78
*CYP26B1*	Cytochrome P450, family 26, subfamily B, polypeptide 1	−1.78
*SLPI*	Secretory leukocyte peptidase inhibitor	−1.78
*PI15*	Peptidase inhibitor 15	−1.78
*RBP7*	Retinol binding protein 7, cellular	−1.77
*SERPINA3*	Serpin peptidase inhibitor, clade A (α-1 antiproteinase, antitrypsin), member 3	−1.77
*CCDC102B*	Coiled-coil domain containing 102B	−1.75
*MTHFD2*	Methylenetetrahydrofolate dehydrogenase (NADP+ dependent) 2, methenyltetrahydrofolate cyclohydrolase	−1.74
*CFI*	Complement factor I	−1.74
*FCGBP*	Fc fragment of IgG binding protein	−1.73
*GPNMB*	Glycoprotein (transmembrane) NMB	−1.73
*FCGR2A*	Fc fragment of IgG, low affinity IIa, receptor (CD32)	−1.72
*MAL2*	Mal, T-cell differentiation protein 2	−1.72
*UAP1*	UDP-*N*-acteylglucosamine pyrophosphorylase 1	−1.71
*IER3*	Immediate early response 3	−1.70
*COL4A1*	Collagen, type IV, α 1	−1.69
*EFNB2*	Ephrin-B2	−1.69
*FCGR2B*	Fc fragment of IgG, low affinity IIb, receptor (CD32)	−1.69
*BTBD3*	BTB (POZ) domain containing 3	−1.68
*FGF13*	Fibroblast growth factor 13	−1.68
*GALNT3*	UDP-*N*-acetyl-α-d-galactosamine:polypeptide *N*-acetylgalactosaminyltransferase 3 (GalNAc-T3)	−1.67
*INHBB*	inhibin, β B	−1.66
*MANSC1*	MANSC domain containing 1	−1.65
*DSP*	Desmoplakin	−1.64
*CLDN8*	Claudin 8	−1.64
*TUBB2B*	Tubulin, β 2B	−1.64
*PODXL*	Podocalyxin-like	−1.63
*EHF*	ETS homologous factor	−1.63
*TIPARP*	TCDD-inducible poly(ADP-ribose) polymerase	−1.63
*ANGPT2*	Angiopoietin 2	−1.62
*ADAMTS1*	ADAM metallopeptidase with thrombospondin type 1 motif, 1	−1.62
*GPR4*	G protein-coupled receptor 4	−1.61
*DBH*	Dopamine β-hydroxylase (dopamine β-monooxygenase)	−1.61
*GPR183*	G protein-coupled receptor 183	−1.61
*TFAP2A*	Transcription factor AP-2 α (activating enhancer binding protein 2 α)	−1.60
*SNORD89*	Small nucleolar RNA, C/D box 89	−1.60
*CXCL2*	Chemokine (C-X-C motif) ligand 2	−1.60
*CXADR*	Coxsackie virus and adenovirus receptor	−1.60
*TPRKB*	TP53RK binding protein	−1.60
*ETS2*	v-ets erythroblastosis virus E26 oncogene homolog 2 (avian)	−1.60
*RAPH1*	Ras association (RalGDS/AF-6) and pleckstrin homology domains 1	−1.60
*ADGRF5*	Adhesion G protein-coupled receptor F	−1.60
*CA2*	Carbonic anhydrase II	−1.59
*LIPA*	Lipase A, lysosomal acid, cholesterol esterase	−1.59
*PGM2*	Phosphoglucomutase 2	−1.59
*KRT19*	Keratin 19	−1.58
*MGAT5*	Mannosyl (α-1,6-)-glycoprotein β-1,6-*N*-acetyl-glucosaminyltransferase	−1.58
*NCF2*	Neutrophil cytosolic factor 2	−1.57
*RHOU*	Ras homolog gene family, member U	−1.57
*ALCAM*	Activated leukocyte cell adhesion molecule	−1.57
*LRRN1*	Leucine-rich repeat neuronal 1	−1.57
*OLR1*	Oxidized low-density lipoprotein (lectin-like) receptor 1	−1.55
*SLC19A2*	Solute carrier family 19 (thiamine transporter), member 2	−1.55
*PRPS2*	Phosphoribosyl pyrophosphate synthetase 2	−1.55
*MEGF10*	Multiple EGF-like domains 10	−1.55
*CYYR1*	Cysteine/tyrosine-rich 1	−1.54
*PLVAP*	Plasmalemma vesicle-associated protein	−1.54
*TM4SF1*	Transmembrane 4 L6 family member 1	−1.54
*PDGFA*	Platelet-derived growth factor α polypeptide	−1.54
*YBX2*	Y box binding protein 2	−1.54
*ATP2B1*	ATPase, Ca^2+^-transporting, plasma membrane 1	−1.54
*PCDHB2*	Protocadherin β 2	−1.54
*DNMT1*	DNA (cytosine-5-)-methyltransferase 1	−1.54
*S100A8*	S100 calcium binding protein A8	−1.53
*MAP2*	Microtubule-associated protein 2	−1.53
*ARRDC4*	Arrestin domain containing 4	−1.52
*FAM83D*	Family with sequence similarity 83, member D	−1.52
*LSR*	Lipolysis stimulated lipoprotein receptor	−1.52
*STK26*	Serine/threonine protein kinase 26	−1.51
*MIR181A2HG*	MIR181A2 host gene (non-protein coding)	−1.51
*VWA8*	von Willebrand factor A domain containing 8	−1.51
*MEST*	Mesoderm-specific transcript homolog (mouse)	−1.51
*ZNF835*	Zinc finger protein 835	1.51
*NAT1*	*N*-acetyltransferase 1 (arylamine *N*-acetyltransferase)	1.51
*EPSTI1*	Epithelial stromal interaction 1 (breast)	1.51
*LOC221946*	Hypothetical LOC221946	1.51
*OAS1*	2′,5′-oligoadenylate synthetase 1, 40/46 kDa	1.52
*SELL*	Selectin L	1.52
*COX6C*	Cytochrome c oxidase subunit VIc	1.52
*TRIM41*	Tripartite motif-containing 41	1.52
*IFI27*	Interferon-α-inducible protein 27	1.52
*IGF1*	Insulin-like growth factor 1 (somatomedin C)	1.52
*SCAMP1-AS1*	SCAMP1 antisense RNA 1	1.52
*CD207*	CD207 molecule, langerin	1.52
*IFI35*	Interferon-induced protein 35	1.52
*GGH*	γ-Glutamyl hydrolase (conjugase, folylpolygammaglutamyl hydrolase)	1.52
*NOX4*	NADPH oxidase 4	1.53
*CNTN3*	Contactin 3 (plasmacytoma associated)	1.53
*CCL5*	Chemokine (C-C motif) ligand 5	1.54
*GALNT1*	UDP-*N*-acetyl-α-d-galactosamine:polypeptide *N*-acetylgalactosaminyltransferase 1 (GalNAc-T1)	1.54
*SPON1*	Spondin 1, extracellular matrix protein	1.54
*SEMA3C*	Sema domain, immunoglobulin domain (Ig), short basic domain, secreted, (semaphorin) 3C	1.54
*DDX60L*	DEAD (Asp-Glu-Ala-Asp) box polypeptide 60-like	1.55
*TNFSF10*	Tumor necrosis factor (ligand) superfamily, member 10	1.55
*CXCL14*	Chemokine (C-X-C motif) ligand 14	1.55
*WISP2*	WNT1 inducible signaling pathway protein 2	1.55
*STAT1*	Signal transducer and activator of transcription 1, 91 kDa	1.55
*COMP*	Cartilage oligomeric matrix protein	1.56
*IGLJ3*	Immunoglobulin lambda joining 3	1.56
*LRRC17*	Leucine-rich repeat containing 17	1.56
*IFI44*	Interferon-induced protein 44	1.56
*ISG15*	ISG15 ubiquitin-like modifier	1.56
*FBLN2*	Fibulin 2	1.57
*SLC6A6*	Solute carrier family 6 (neurotransmitter transporter, taurine), member 6	1.57
*MX2*	Myxovirus (influenza virus) resistance 2 (mouse)	1.57
*SH3D19*	SH3 domain containing 19	1.57
*TRBC1*	T-cell receptor β constant 1	1.58
*SGCE*	Sarcoglycan, epsilon	1.58
*IGHM*	Immunoglobulin heavy constant mu	1.58
*DCBLD1*	Discoidin, CUB and LCCL domain containing 1	1.59
*PPAPDC1A*	Phosphatidic acid phosphatase type 2 domain containing 1A	1.59
*BST2*	Bone marrow stromal cell antigen 2	1.59
*MFAP2*	Microfibrillar-associated protein 2	1.60
*PDGFD*	Platelet-derived growth factor D	1.60
*IGKC*	Immunoglobulin kappa constant	1.60
*CST1*	Cystatin SN	1.61
*CCL8*	Chemokine (C-C motif) ligand 8	1.61
*RASGRF2*	Ras protein-specific guanine nucleotide-releasing factor 2	1.61
*MX1*	Myxovirus (influenza virus) resistance 1, interferon-inducible protein p78 (mouse)	1.63
*PDGFRL*	Platelet-derived growth factor receptor-like	1.63
*ALDH1L2*	Aldehyde dehydrogenase 1 family, member L2	1.63
*FAM198B*	Family with sequence similarity 198, member B	1.63
*MIR100HG*	Mir-100-let-7a-2 cluster host gene	1.64
*GAPT*	GRB2-binding adaptor protein, transmembrane	1.65
*SELM*	Selenoprotein M	1.65
*DSCAM-AS1*	DSCAM antisense RNA 1	1.66
*STMN2*	Stathmin-like 2	1.69
*FBLN5*	Fibulin 5	1.70
*IFIT3*	Interferon-induced protein with tetratricopeptide repeats 3	1.70
*SFRP4*	Secreted frizzled-related protein 4	1.71
*ACKR4*	Atypical chemokine receptor 4	1.71
*CPNE2*	Copine II	1.71
*PSMB9*	Proteasome (prosome, macropain) subunit, β type, 9 (large multifunctional peptidase 2)	1.72
*ST6GAL2*	ST6 β-galactosamide α-2,6-sialyltranferase 2	1.72
*NEXN*	Nexilin (F actin binding protein)	1.72
*CD52*	CD52 molecule	1.72
*MFAP5*	Microfibrillar associated protein 5	1.73
*RARRES3*	Retinoic acid receptor responder (tazarotene induced) 3	1.75
*GXYLT2*	Glucoside xylosyltransferase 2	1.75
*HMCN1*	Hemicentin 1	1.76
*EFEMP1*	EGF-containing fibulin-like extracellular matrix protein 1	1.78
*IL21R*	Interleukin 21 receptor	1.78
*C8orf4*	Chromosome 8 open reading frame 4	1.78
*LINC01503*	Long intergenic non-protein coding RNA 1503	1.78
*OLFML3*	Olfactomedin-like 3	1.79
*CILP*	Cartilage intermediate layer protein, nucleotide pyrophosphohydrolase	1.81
*MVB12A*	Multivesicular body subunit 12A	1.82
*SCUBE2*	Signal peptide, CUB domain, EGF-like 2	1.83
*WNT2*	Wingless-type MMTV integration site family member 2	1.85
*APOL3*	Apolipoprotein L3	1.87
*ADRA2A*	Alpha-2A adrenergic receptor	1.89
*HIST1H3I*	Histone cluster 1, H3i	1.92
*SLC46A3*	Solute carrier family 46, member 3	1.92
*ARHGAP28*	Rho GTPase activating protein 28	1.93
*KANK4*	KN motif and ankyrin repeat domains 4	1.93
*SDC1*	Syndecan 1	1.95
*CMPK2*	Cytidine monophosphate (UMP-CMP) kinase 2, mitochondrial	1.96
*IFI44L*	Interferon-induced protein 44-like	1.97
*FMO1*	Flavin containing monooxygenase 1	1.98
*TMEM119*	Transmembrane protein 119	1.99
*FNDC1*	Fibronectin type III domain containing 1	2.00
*ADAMDEC1*	ADAM-like, decysin 1	2.00
*TPSAB1*	Tryptase α/β1	2.02
*CPA3*	Carboxypeptidase A3 (mast cell)	2.02
*MMP3*	Matrix metallopeptidase 3 (stromelysin 1, progelatinase)	2.05
*IFI6*	Interferon, α-inducible protein 6	2.06
*IFIT1*	Interferon-induced protein with tetratricopeptide repeats 1	2.06
*SFRP2*	Secreted frizzled-related protein 2	2.09
*TRIM6*	Tripartite motif-containing 6	2.10
*TPSB2*	Tryptase β2 (gene/pseudogene)	2.19
*RSAD2*	Radical *S*-adenosyl methionine domain containing 2	2.28
*LOXL1*	Lysyl oxidase-like 1	2.30
*OMD*	Osteomodulin	2.35
*IGJ*	Immunoglobulin J polypeptide, linker protein for immunoglobulin α and mu polypeptides	2.44
*FCGR1A*	Fc fragment of IgG, high affinity Ia, receptor (CD64)	2.47
*MATN3*	Matrilin 3	2.55
*IGLV@*	Immunoglobulin lambda variable cluster	2.65
*OGN*	Osteoglycin	2.99
*EPYC*	Epiphycan	3.04

Negative values for fold change indicate up-regulation in older patient samples; positive fold change values indicate up-regulation in younger patient samples

### Data validation

**Fig. 4:**
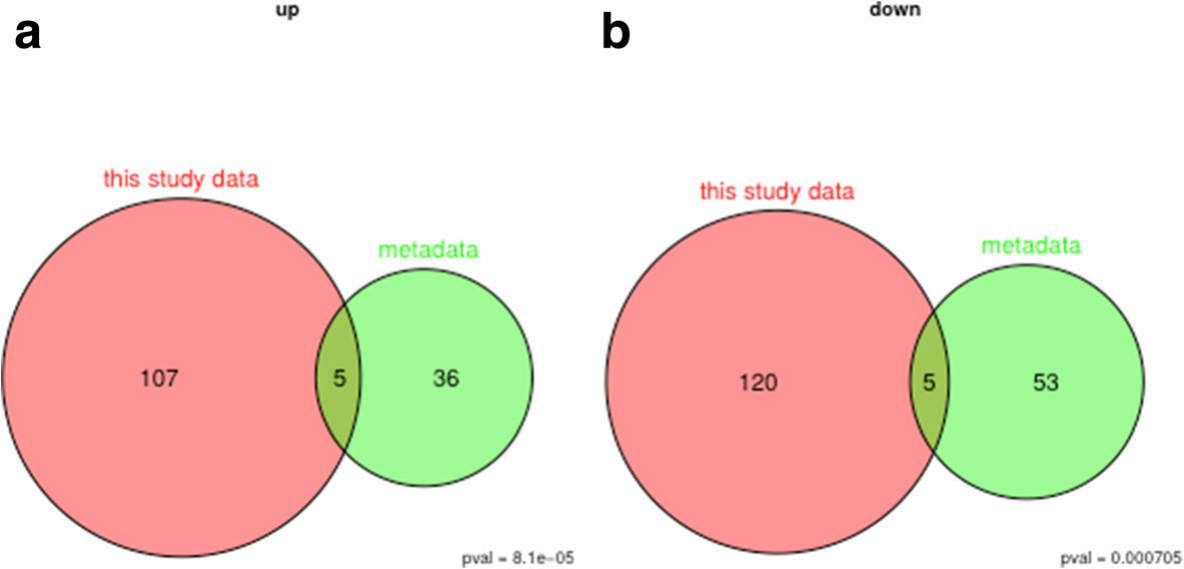
Venn diagrams showing the intersection of our own expression data (‘study data’) and the publicly available data (‘metadata’). **a** The up-regulated genes in young stromal samples. **b** The down-regulated genes in young stromal samples

We used publicly available data sets ([GEO:GSE5847] [9], 34 samples; [GEO:GSE4823] [7], 33 samples; [GEO:GSE14548] [8], 9 samples) to validate our findings because of the limited size of our study group. We found a significant overlap for ten genes, of which five showed higher expression in older patients (*p* < 0.01) and five showed lower expression in older patients (*p* < 0.01). Venn diagrams depicting the overlapping genes are shown in Fig. 4; gene details are listed in Table 3.

**Table 3:** Significant up- or down-regulated genes after validation in the external validation data set (*see* Fig. 4)

Gene	Full name	Fold change
*RARRES3*	Retinoic acid receptor responder (tazarotene induced) 3	1.75
*SFRP4*	Secreted frizzled-related protein 4	1.71
*SCUBE2*	Signal peptide, CUB domain, EGF-like 2	1.83
*NAT1*	*N*-acetyltransferase 1 (arylamine *N*-acetyltransferase)	1.51
*COMP*	Cartilage oligomeric matrix protein	1.56
*ANXA3*	Annexin A3	−2.06
*PROM1*	Prominin 1	−2.27
*FGF13*	Fibroblast growth factor 13	−1.68
*TUBB2B*	Tubulin, beta 2B	−1.64
*WIF1*	WNT inhibitory factor 1	−2.10

Negative values for fold change indicate up-regulation in old patient samples, and positive values indicate up-regulation in young patient samples

### Gene set enrichment analysis

**Fig. 5:**
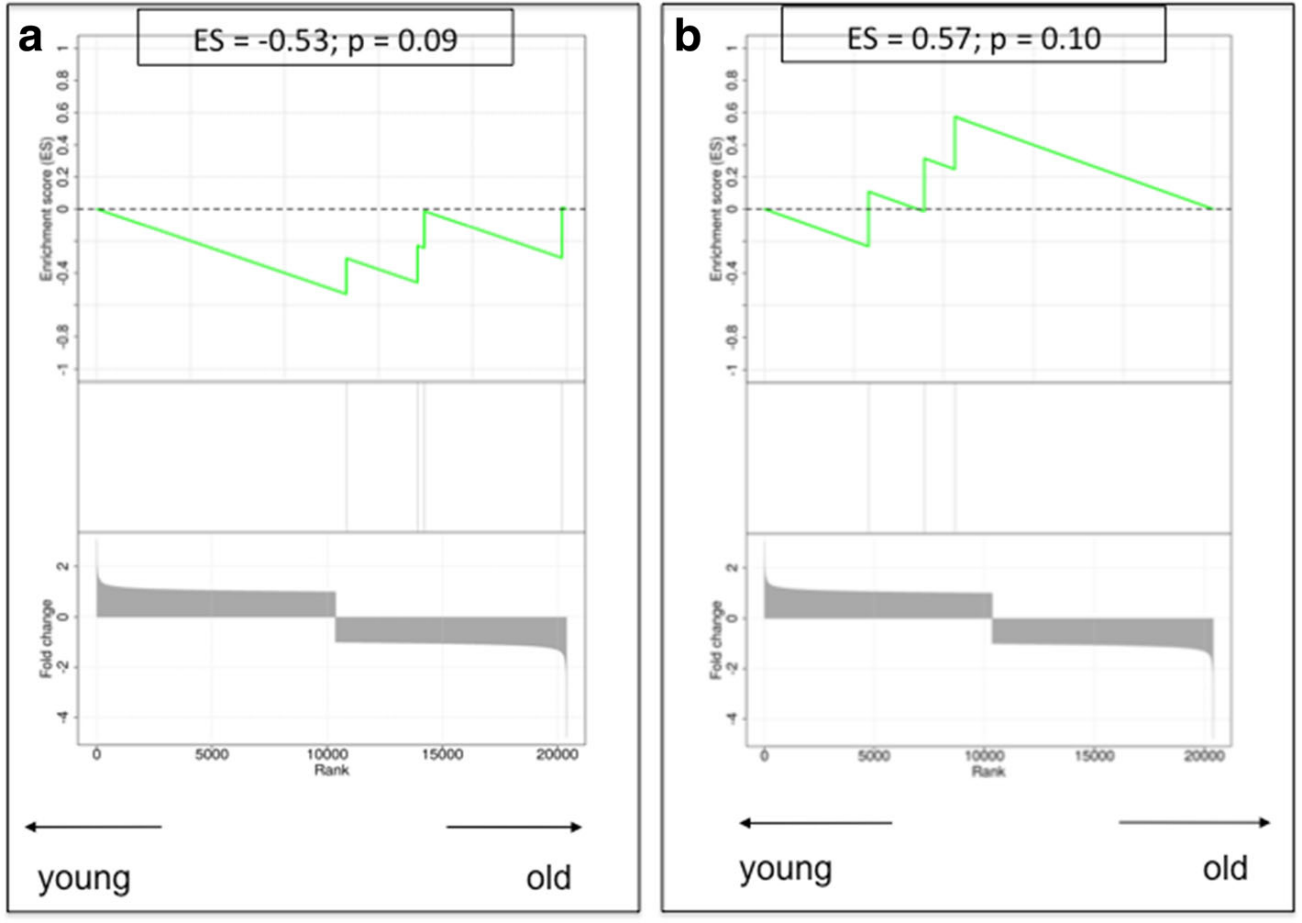
Gene set enrichment analysis plots. **a** Senescence genes. **b** The DNA damage response process Low values correspond to enrichment of the genes in older patients, and high values correspond to enrichment of the genes in younger patients. *ES* Enrichment score

**Fig. 6:**
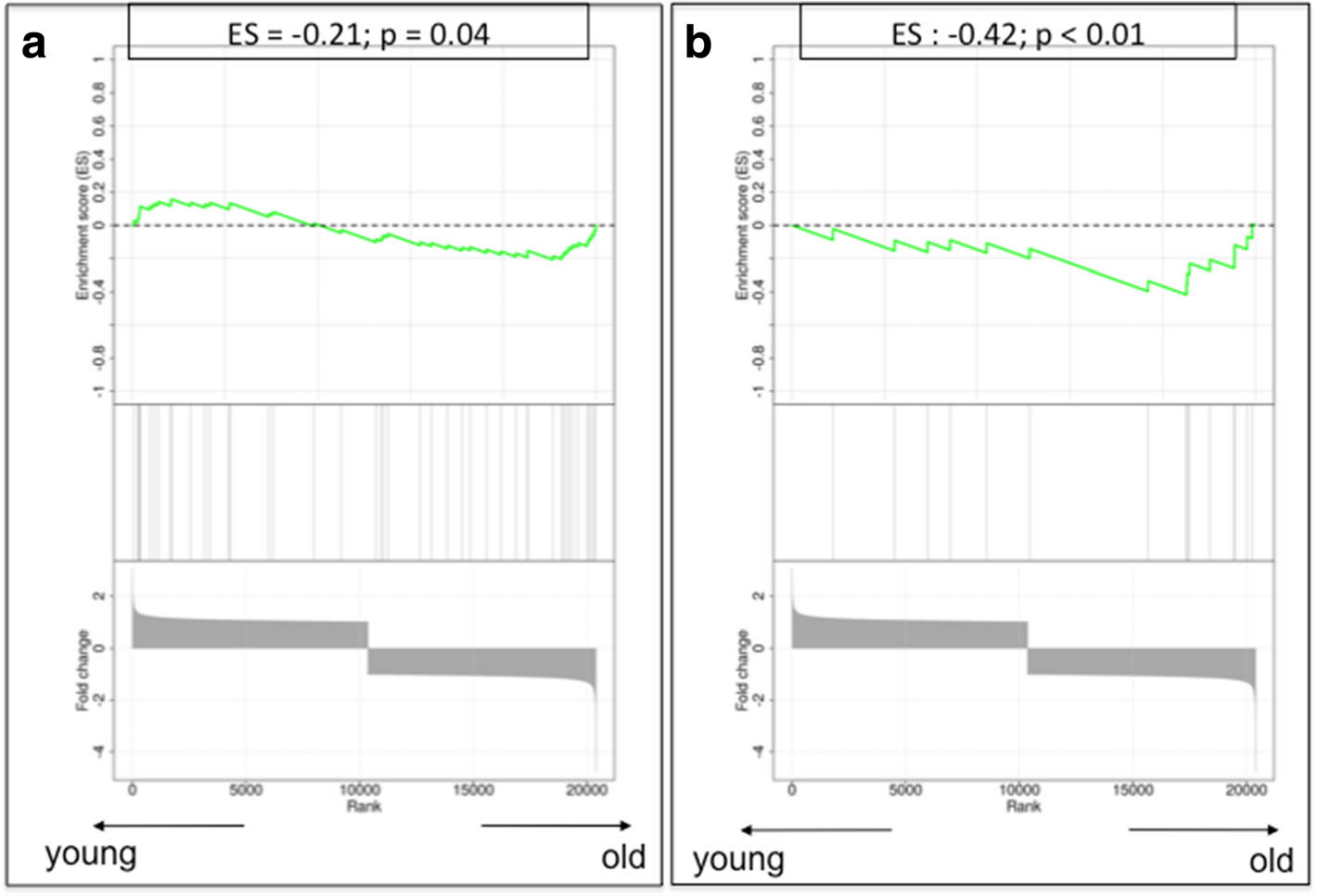
Gene set enrichment analysis plots. **a** The senescence-associated secretory profile process. **b** The autophagy-to-senescence transition or ‘reverse Warburg effect’. Low values correspond to enrichment of the genes in older patients, and high values correspond to enrichment of the genes in younger patients. *ES* Enrichment score

Next, we performed GSEA to measure the expression of pre-defined gene sets related to specific biological processes. The resulting ES, which ranges from −1 to 1, reflects the enrichment in genes of a given reference class among the top up- or down-regulated genes from the individual gene ranking. Plots are shown in Figs. 5 and 6. The genes that were included in each GSEA, with respective literature references, are listed in Table 4.

**Table 4:** Groups of candidate genes related to a specific pathophysiological process, built to perform gene set enrichment analysis, and their respective references

Gene group	Involved genes	References
Cellular senescence	*CDKN1A*, *CDKN2A*, *TP53*, *RB1*, *GLB1*	[17–19, 69–71]
DNA damage response	*ATM*, *NBN*, *CHEK2*	[21, 73]
Senescence-associated secretory profile	*IL1A*, *IL6*, *IL6R*, *IL6ST*, *IL8*, *CXCL1*, *CXCL2*, *CXCL3*, *CSF2*, *IL7*, *ICAM1*, *TNFRSF11B*, *HGF*, *IGFBP4*, *CCL8*, *PLAUR*, *IGFBP2*, *CCL26*, *IL13*, *CCL20*, *ICAM3*, *PGF*, *TNFRSF1A*, *TNFRSF1B*, *CCL13*, *CCL16*, *TNFRSF10C*, *CCL2*, *FAS*, *ANG*, *IGFBP6*, *IL1B*, *(CCL3)*, *TIMP2*, *IL11*, *OSM*, *LEP*, *AXL*, *KITLG*, *FGF7*, *IL15*, *FGF2*, *IGFBP1*, *MIF*	[17, 21, 22, 26]
Autophagy-to-senescence transition	*CAV1*, *CTSB*, *BNIP3*, *PRKAA1*, *PRKAA2*, *LAMP2*, *MAP1LC3B*, *ATG16L1*, *HIF1A*, *NFKB1*, *DRAM1*, *TP73*, *MAPK8*, *E2F1*, *STK11*	[33–37]

### Senescence genes

In the individual gene expression analysis, no significant difference was found for genes known to be associated with senescence, such as *CDKN1A*, *CDKN2A*, *TP53*, *GLB1* or the retinoblastoma (*RB*) genes. Nevertheless, the enrichment analysis for this gene set resulted in an ES of −0.53, suggesting enrichment of senescence genes in the stroma of older patients, although statistical significance was not reached (*p* = 0.09) (Fig. 5a). The lack of significance might be due to the small sample size of the reference classes.

### DNA damage response

None of the three most important components of the DNA damage response, namely *ATM*, *NBN* (*NBS1*) and *CHK2*, were differentially expressed between young and old stromal tissues. Similar results were found when we applied GSEA to the DNA damage response gene set. The estimated gene score was 0.57, which did not reach statistical significance (*p* = 0.10) (Fig. 5b).

### Senescence-associated secretory profile

Several genes involved in the SASP showed a deregulated gene expression profile, suggesting an enrichment of SASP in the stroma of older patients, including *CXCL2* (over-expressed in older stromal tissues; fold change 1.59), *TNFRSF11B* (over-expressed in older stromal tissues; fold change 2.11) and *CCL8* (down-regulated in older stromal tissues; fold change −1.61). Of interest, GSEA confirmed a significant enrichment in SASP-related genes within the stroma of older patient samples with an ES of −0.21 (*p* = 0.04) (Fig. 6a).

### The reverse Warburg effect: autophagy genes

None of the genes described to be involved in the AST showed a relevant difference in gene expression between young and old stroma at the individual gene level. However, when compiling them together in the GSEA, we found a highly significant enrichment of autophagy genes in the stroma of older patient samples (ES −0.42; *p* < 0.01) (Fig. 6b).

## Discussion

The reason for the age-related increase in cancers has been debated for decades. Besides cumulative DNA damage throughout life, the accumulation of senescent cells is assumed to create a tumour-promoting micro-environment through phenomena such as the SASP and the AST. So far, studies investigating the impact of stromal senescence on tumour development have been based on in vitro fibroblast cultures where senescence was artificially induced [23, 29]. As a consequence of this approach, an overload of senescent or pre-senescent fibroblasts was present in these experiments. We do not know if this accurately reflects the situation in spontaneous cancers. Accumulation of senescent cells with age has been studied mostly in fibroblasts localised in the skin [14], but data on the frequency of senescent fibroblasts in the older breast are lacking. Moreover, controversy exists regarding whether findings on in vitro senescence can be extrapolated to the situation in vivo. We therefore aimed in this study to investigate the molecular footprint of the older breast cancer micro-environment in order to support in vivo confirmation of key concepts such as ageing/senescence, DNA damage response, SASP and AST. Senescence in the surrounding stroma is expected to result in a pro-tumourigenic micro-environment with stimulation of proliferation, migration/invasion and de-differentiation. However, this had never been shown in spontaneously occurring breast cancers. For this purpose, we selected two groups of patients with triple-negative breast cancer belonging to extreme age categories, isolated cancer-associated stromal fields via LCM and investigated their gene expression profiles.

Differential gene expression analysis using a cut-off of a 1.5-fold change in expression revealed 120 up-regulated and 107 down-regulated genes in the stromal parts of older patients compared with the younger patients. Validation of these findings using publicly available stromal data revealed a set of ten differentially expressed genes between young and old stromal samples. The young stromal samples showed mainly up-regulation in genes such as *RARRES3*, *SCUBE2*, *SFRP4*, *COMP* and *NANT1* that preclude migration and invasion by stabilising the cells in the extracellular matrix and stimulate differentiation [41, 51–60]. Significant up-regulation in the older stromal micro-environment was shown instead for genes that are involved in proliferation, de-differentiation and angiogenesis. Four genes, namely *ANXA3*, *PROM1*, *FGF13* and *TUBB2B*, seem to restrain differentiation and promote cell proliferation, invasiveness and metastasis [61–71]. The fifth up-regulated gene in the older stromal samples, *WIF1*, is a negative inhibitor of the Wnt (Wingless-type)/β-catenin signalling pathway. It is thought to inhibit proliferation and to induce differentiation and cellular senescence by up-regulation of tumour suppressor genes such as *TP53* or *P21* [72, 73]. Although the proliferation-inhibiting and differentiation-inducing effects of this gene seem to be in contradiction with the proliferation- and metastasis-promoting activity of the other four up-regulated genes in the older stromal samples, its senescence-inducing function may be an obvious explanation for the age-related stromal expression of *WIF1* in our study. Taken together, on the basis of our data, we found evidence of a more tumour-favourable micro-environment in the stromal samples from older patients than in those from younger patients.

As an additional analysis, we applied a candidate gene approach by assembling sets of genes on the basis of the literature. We specifically looked at the individual gene expression results for these genes, but we also compiled them using a gene set enrichment strategy that reflects the representation of these genes among the top up- or down-regulated genes in old and young stromal samples.

The molecular process of senescence is characterised by up-regulation of several senescence genes. The most documented ones are *TP53*, *CDKN2A* (*P16*), *CDKN1A* (*P21*) and *RB* [74–76]. These major senescence-inducing genes did not show significantly different expression values between young and old stroma in the individual gene analysis. Nevertheless, we observed in older patients an up-regulation of *PAI-1* (*SERPINE1*), a matrix-remodelling enzyme, which has also been described as a crucial regulator of ageing and senescence by acting downstream of *TP53* and upstream of insulin-like growth factor binding protein 3 [77], and of *WIF1*, described above as an inhibitor of the Wnt/β-catenin signalling pathway and an inducer of senescence, and it was also significantly increased in samples from old compared with young patients (Table 2).

These findings could be indicative of more widespread cellular senescence in our older stromal samples compared with the young ones. Gene enrichment analysis based on five key senescence genes, including the ones mentioned above, showed a tendency towards a more prominent senescence trait in older stroma, but no significant ES was reached for this process in the older patient samples. Therefore, we cannot decisively conclude that samples from the older patient group show increased senescence.

The DNA damage response is a biological process that, upon severe DNA damage, triggers the switch towards a permanent growth arrest [78]. It was found that the molecular senescence programme can be induced only when this DNA damage response has been activated for a sufficiently long time period [21]. We could not demonstrate any clear difference in the individual expression of three key players involved in the DNA damage response (*ATM*, *NBN* or *CHEK2*) [78], nor did we find significant enrichment for this set of genes in the older patient samples.

In our stromal gene expression study, only a few of the SASP components described by Coppé et al. as overproduced by senescent cell cultures [26] showed significant age-related differential expression: *CXCL2* and osteoprotegerin (*TNFRSF11B*), a member of the tumour necrosis factor receptor superfamily, which both showed overexpression in the old patient samples, as well as *CCL8*, which showed down-regulation in the older versus the younger stromal samples. Of interest, when compiling all the components of the SASP together in the gene enrichment analysis, we indeed confirmed a significant enrichment in SASP genes among genes up-regulated in the older patient samples, confirming for the first time the presence of the SASP phenotype in human breast cancers in vivo.

Autophagy is assumed to precede or parallel the process of senescence [38], as described by the term *autophagy-to-senescence transition* (AST). Typical markers for AST are loss of caveolin 1 (*CAV1*) and up-regulation of *BNIP3*, *BNIP3L*, Beclin-1, Cathepsin B and *ATG16L1*. Our individual gene expression results did not show relevant up-regulation of single autophagy-related genes in the older stromal samples, but compilation of these genes into a GSEA showed highly significant enrichment for autophagy genes in the older stromal samples. Thus, in addition to the presence of SASP, we also confirmed the presence of AST in the older stromal cancer milieu.

In this study, regarding gene expression levels, we report, for the first time to our knowledge, the presence of SASP and AST in older stromal samples, supporting the previously published in vitro and xenograft findings. This does not, however, solve the paradox between the stimulatory effect that these processes are supposed to have on proximate malignant cells and the clinical finding that breast cancer in older patients behaves in a rather more indolent instead of a more aggressive way [79]. Also, it remains puzzling that we found evidence for SASP and AST in older stromal samples, which are senescence-related phenomena, whereas we did not find convincing evidence for increased senescence in these samples. The small sample size, together with the low number of genes defining the ‘senescence’ programme, could partly explain the lack of significance for major senescence-related genes such as *TP53*, *CDKN2A* and *pRB*, both at the individual level and in the GSEAs.

Besides the small sample size of the study, the broad age interval between the patient groups and the difference in menopausal status between the groups introduce other potential biases, because not only the stroma but also infiltrating immune cells are believed to be altered by hormonal changes. Ideally, our findings would be further investigated in larger patient cohorts including other age categories, and validated by proteomic analysis of the stromal tissue. LCM is a demanding technique, however, limiting the number of patient samples that can be processed. We attempted to compensate for some of these limitations by including a validation strategy on publicly available gene expression data.

## Conclusions

We report, for the first time to our knowledge, the involvement of key pathophysiological concepts of cancer and ageing, such as the SASP and the AST, in vivo in human cancer patients. These remarkable findings justify further research to fully elucidate the role of the ageing stroma in (breast) tumour development and progression. In the first place, this research should be extended in other subtypes of breast cancer and more age categories.

## Additional file

Additional file 1: Table S1: RNA concentration and RNA Quality Indicator value before, and RNA concentration after, amplification. (DOCX 14 kb)

## Abbreviations

AST: Autophagy-to-senescence transition
CAV1: Caveolin 1
ER: Oestrogen receptor
ES: Enrichment score
fRMA: Frozen robust multi-array analysis
GEO: Gene Expression Omnibus
GSEA: Gene set enrichment analysis
H&E: Haematoxylin and eosin
HER2: Human epidermal growth factor receptor 2
LCM: Laser capture microdissection
MMP3: Matrix metalloproteinase 3
NCBI: National Center for Biotechnology Information
PR: Progesterone receptor
RB: Retinoblastoma
SASP: Senescence-associated secretory profile
SPIA: Single-primer isothermal amplification
