# Supplementary material for: The footprint of the ageing stroma in older patients with breast cancer
Source: Breast Cancer Res. 2017 Jul 3;19:78. doi: 10.1186/s13058-017-0871-0 (PMC5494807; doi:10.1186/s13058-017-0871-0)
Supplement: Additional file 1: Table S1: — RNA concentration and RNA Quality Indicator value before, and RNA concentration after, amplification. (DOCX 14 kb) [file 13058_2017_871_MOESM1_ESM.docx]

| Patient ID | RNA concentration before amplification (ng/microliter) | RQI | RNA concentration after amplification  (ng/microliter) |
| --- | --- | --- | --- |
| 1 ^$^ | 4,6 | 3,9 | 340,8 |
| 2 ^$^ | 7,7 | 6,9 | 441,7 |
| 3 ^$^ | 3,4 | na | 498,8 |
| 4 ^$^ | 9,2 | 4,6 | 361,8 |
| 5 ^$^ | 7,2 | 5 | 328,9 |
| 6 ^$^ | 3,8 | 4,8 | 344,5 |
| 7 ^$^ | 5,8 | 6,7 | 439,2 |
| 8 ^$^ | 4,9 | 2,2 | 410,8 |
| 9 ^$^ | 3,9 | na | 388,3 |
| 10 ^δ^ | 12,3 | 7,7 | 458,6 |
| 11 ^δ^ | 9,7 | 6 | 482,0 |
| 12 ^δ^ | 10,7 | 6,5 | 577,1 |
| 13 ^δ^ | 6,6 | 6,6 | 338,9 |
| 14 ^δ^ | 7,5 | 6,8 | 346,3 |
| 15 ^δ^ | 8,8 | 3,9 | 266,2 |
| 16 ^δ^ | 4,5 | 7,0 | 448,7 |
| 17 ^δ^ | 8,0 | 2,7 | 465,0 |

^$^ patient belongs to the young patient group

^δ^ patient belongs to the older patient group

na: not available

Additional file 1: **Table S1**: RNA concentration and RQI value (RNA Quality Indicator) before, and RNA concentration after amplification
